# The *Dictyostelium discoideum* RACK1 orthologue has roles in growth and development

**DOI:** 10.1186/1478-811X-12-37

**Published:** 2014-06-15

**Authors:** Napoleon Nosa Omosigho, Karthic Swaminathan, Markus Plomann, Annette Müller-Taubenberger, Angelika A Noegel, Tanja Y Riyahi

**Affiliations:** 1 Institute of Biochemistry I, Medical Faculty, Center for Molecular Medicine Cologne (CMMC) and Cologne Excellence Cluster on Cellular Stress Responses in Aging-Associated Diseases (CECAD), University of Cologne, 50931 Köln, Germany; 2 Institute of Biochemistry II, Medical Faculty, University of Cologne, 50931 Köln, Germany; 3 Institute of Anatomy and Cell Biology, Ludwig-Maximilians-University, 80336 München, Germany

**Keywords:** *Dictyostelium discoideum*, G protein signaling, RACK1, WD40 repeat protein, Phosphoinositides, Phosphorylation, Dimerization

## Abstract

**Background:**

The receptor for activated C-kinase 1 (RACK1) is a conserved protein belonging to the WD40 repeat family of proteins. It folds into a beta propeller with seven blades which allow interactions with many proteins. Thus it can serve as a scaffolding protein and have roles in several cellular processes.

**Results:**

We identified the product of the *Dictyostelium discoideum gpbB* gene as the *Dictyostelium* RACK1 homolog. The protein is mainly cytosolic but can also associate with cellular membranes. DdRACK1 binds to phosphoinositides (PIPs) in protein-lipid overlay and liposome-binding assays. The basis of this activity resides in a basic region located in the extended loop between blades 6 and 7 as revealed by mutational analysis. Similar to RACK1 proteins from other organisms DdRACK1 interacts with G protein subunits alpha, beta and gamma as shown by yeast two-hybrid, pulldown, and immunoprecipitation assays. Unlike the *Saccharomyces cerevisiae* and *Cryptococcus neoformans* RACK1 proteins it does not appear to take over Gβ function in *D. discoideum* as developmental and other defects were not rescued in Gβ null mutants overexpressing GFP-DdRACK1. Overexpression of GFP-tagged DdRACK1 and a mutant version (DdRACK1mut) which carried a charge-reversal mutation in the basic region in wild type cells led to changes during growth and development.

**Conclusion:**

DdRACK1 interacts with heterotrimeric G proteins and can through these interactions impact on processes specifically regulated by these proteins.

## Background

Every cell has the capability to detect extracellular signals, and then mounts an appropriate response to these signals. Specific stimuli include light, hormones, neurotransmitters, growth factors, and odorants. They are sensed by cell surface receptors which in case of G protein-coupled receptors interact with heterotrimeric guanine nucleotide binding proteins (G proteins), key intermediates in cellular signaling processes that link the receptors with intracellular effector proteins generating cellular responses [1,2].

The heterotrimeric G proteins consist of α, β, and γ subunits. Upon binding of agonist to the receptor, a conformational change in the Gα subunit promotes the release of GDP and binding to GTP which then releases Gβγ [2]. The liberated Gβγ subunits play critical roles in many cellular processes [3,4]. They regulate a variety of effector molecules ranging from enzymes, such as phospholipase Cβ (PLCβ) and adenylyl cyclase, to ion channels. The Gβγ complex functions at many levels to promote and restrict signaling at the plasma membrane. It can act as a guanine nucleotide dissociation inhibitor (GDI) to prevent spontaneous exchange of GTP for GDP on Gα [5]. On the other hand, its activity is regulated by a number of interacting proteins which can also represent effectors. Such proteins are phosducin, phosducin-like proteins, and G protein-coupled receptor kinases (GRKs) [6,7]. Gβ subunits adopt a distinct seven-bladed propeller structure with each blade composed of a conserved core of ~40 amino acids flanked by Trp-Asp (WD) [8,9]. Unlike higher eukaryotes in which multiple Gβ subunits have been identified [10], *D. discoideum* harbors a single Gβ, one Gγ, and twelve Gα subunits [11-13]. All the Gα subunits are expected to interact with the same Gβγ dimer. *D. discoideum* development is relatively simple as compared to higher eukaryotes. The roles of its individual Gα subunits, however, appear to be quite distinct with respect to developmental morphology and cellular differentiation as indicated by the phenotypes of gene disruption or overexpression mutants. Gα2 is required for adenylyl cyclase A (ACA), guanylyl cyclase (GC) and phospholipase C (PLC) activation. Gα2-null mutants do not aggregate and overexpression of wild-type Gα2 results in precocious activation of guanylyl cyclase by cAMP in vegetative cells [14]. Gα4 mediates responses to folic acid [15], and Gα8 inhibits proliferation, promotes adhesion and regulates cell differentiation [16]. *D. discoideum* cells lacking functional G protein β subunit are severely defective in phagocytosis, chemotaxis, aggregation, and development [17,12-21].

RACK1 (Receptor for activated C kinase 1) is present in organisms from all eukaryotic kingdoms like plants, fungi and animals. *S. cerevisiae* cells lacking RACK1 are viable whereas in a mouse model RACK1 depletion causes lethality at gastrulation [22]. The protein was originally found in association with activated protein kinase C (PKC) where it acted as a scaffold protein serving as a platform for connecting PKC with its substrates, and was responsible for the association of activated PKC with cellular membranes [23,24]. The mechanism of membrane interaction is poorly understood. One prediction is that the anchoring protein should always be localized to the same site as its interaction partners. For instance, RACK1 accompanies PKCβII to its site of action in response to its activation [24]. RACK1 interacts with many receptors and their precursors and is involved in their localization. Furthermore RACK1 has been shown to interact with subunits of the heterotrimeric G proteins [25-28].

RACK1 structurally mimics a Gβ harboring seven WD repeats which build up the seven-bladed beta-propeller. Different from Gβ RACK1 lacks the typical N-terminal alpha helix which is necessary for the tight interaction of Gβ with the Gγ subunit. In *S. cerevisiae* and *C. neoformans*, which possess only one Gβγ subunit but multiple Gα subunits, RACK1 has been reported to interact with free Gα and Gγ; interactions with the heterotrimeric Gβγ subunits were shown as well [29,30]. Furthermore, RACK1 was found as a part of the ribosome complex and could thereby be involved in protein translation. Thus RACK1 is a versatile and dynamic component which is involved in many cellular processes far more than PKC could mediate [31,32].

Here we show that *D. discoideum* GpbB (DDB0185122) which is described as a Gβ-like protein in the databases is a RACK1 homolog. We initially identified GbpB as a binding partner of RpkA, an unusual G protein coupled receptor (GPCR) which functions in phagocytosis and antibacterial defense in *D. discoideum*[33]. RpkA has a lipid kinase domain at its C-terminus which contains the interaction site for RACK1. We characterized the protein with regard to its dimerization properties, studied its localization and expression during development and possible interactions with G proteins. Furthermore we uncovered a lipid binding property which is mediated by a unique extended basic loop between blades 6 and 7 of the propeller.

## Results

### Characterization of *D. discoideum* RACK1 (DdRACK1)

**Figure 1:**
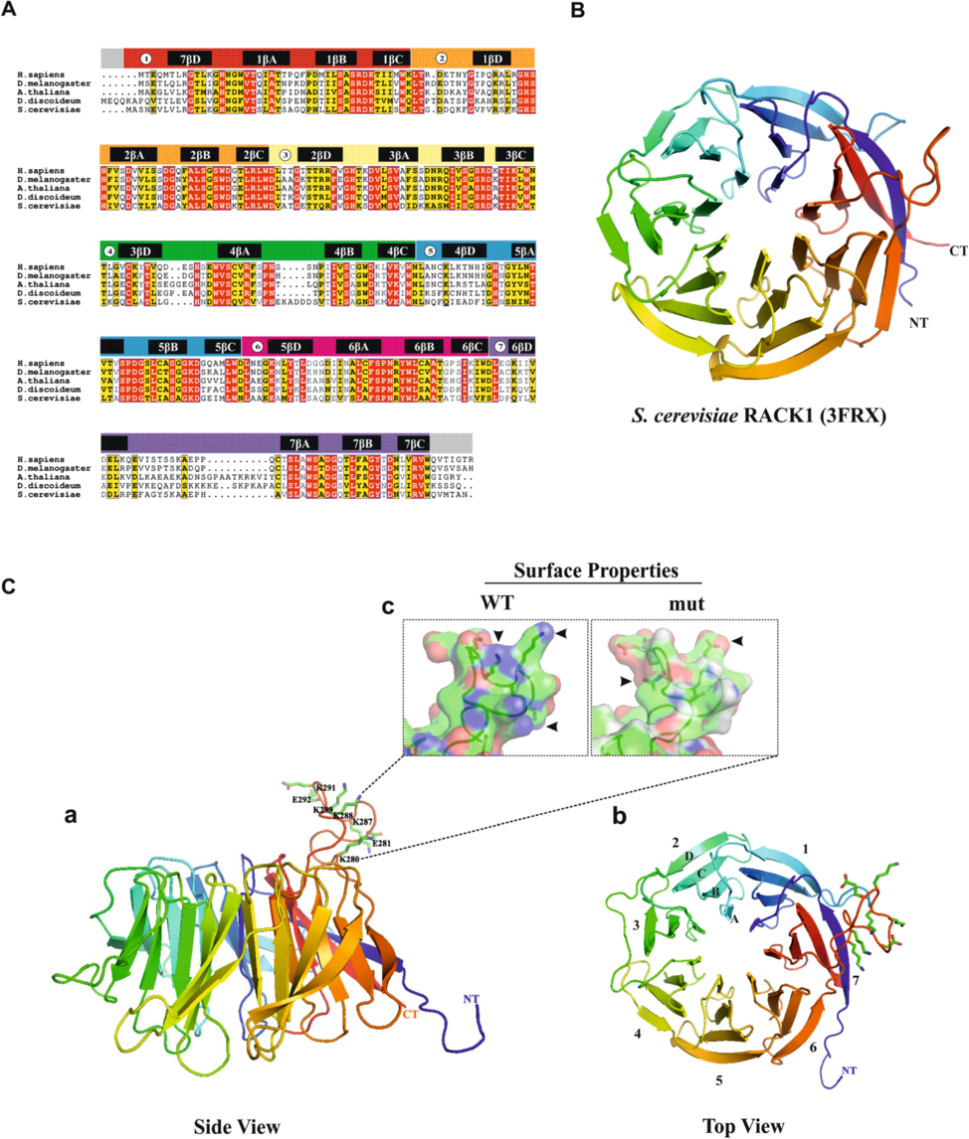
**Structure of RACK1 proteins. (A)** Sequence alignment of RACK1 orthologues and their UniProt accession numbers from *H. sapiens* (P63244), *D. melanogaster* (O18640), *A. thaliana* (O24456), *D. discoideum* (DdRACK1) (P46800) and *S. cerevisiae* (P38011). The WD40 repeats and β-propeller blade positions are written above the sequences. Alignment was done with the ClustalW program and processed through ESPript for representation. All conserved residues are shown in red and similar residues in yellow. **(B)** Ribbon diagram of *S. cerevisiae* RACK1 (Asc1p) (PDB: 3FRX) which was used as template for DdRACK1 model. Shown are the seven β-propeller blades. Coordinates were retrieved from protein data bank (PDB) (http://www.rcsb.org) and modelled with the aid of MODELLER v9 program and visualized by the software PyMOL. **(C)** Ribbon structure of DdRACK1 (DDB0185122) showing (a), the side view of the β-propeller blades with some of the residues in the extended loop between blades 6 and 7 shown in ball and stick model, (b) the top view of the β-propeller blades with the same extended loop as in (a), and (c) the surface properties of DdRACK1 (WT) and DdRACK1mut (mut). Arrowheads pointing to blue and red regions indicate non-mutated and mutated residues, respectively. Structures were modeled and generated with the aid of MODELLER v9 program and visualized using molecular visualization software PyMOL.

gpbB (DDB_G0275045) is located on chromosome 2 of the *D. discoideum* genome and has 2 exons. The open reading frame encompasses 1136 bp which encodes a protein of 329 amino acids migrating as a 36 kDa protein on SDS polyacrylamide gels. Blast results showed that GpbB is highly related to the RACK1 family of proteins and the alignment of RACK1 sequences from diverse organisms such as *H. sapiens*, *D. melanogaster*, *A. thaliana*, *D. discoideum* and *S. cerevisiae* revealed significant sequence identity. The greatest difference is observed between propeller blades 6 and 7 where an extended loop of mainly basic amino acids is present in the *D. discoideum* and the *A. thaliana* RACK1 proteins (Figure 1A).

Gβ was the first WD-repeat protein to be characterized by X-ray crystallography [34]. Since then various other crystal structures have been reported for WD-repeat proteins [35,36] which include the recently determined structures for several RACK1 proteins, RACK1A from *A. thaliana*, Asc1p from *S. cerevisiae*, RACK1 from *T. thermophila* and RACK1 from human [37-41]. These structural studies confirmed the seven-bladed β-propeller structure. In the RACK1 structure each propeller blade consists of a four-stranded antiparallel β-sheet, where strand A lines the central canal of the protein, and strand D is present on the outer circumference. Adjacent blades are connected by a loop bridging from strand D on one blade to strand A on the next. These loops are exposed on the top face of the propeller blade as are the β-turns linking strands B and C in each blade. The loops connecting strand A to B and strand C to D in each blade are located on the reverse, slightly larger face of the propeller [31]. Most notably, the D-A loop between blades 6 and 7 in the RACK1 species is 8 to 19 residues longer than the cognate region of Gβ_1_ and forms a knob-like projection from the upper face of the propeller (discovered in the crystal structure of *A. thaliana* RACK1A) [31]. This sequence is quite unusual in the *D. discoideum* protein as it is rich in lysine residues. The general features described for RACK1 proteins are also present in DdRACK1 when we modelled the DdRACK1 sequence to the crystal structure of *S. cerevisiae* RACK1 (Asc1p) which reveals a comparable structure (Figure 1B, C).

### Subcellular distribution and developmental expression pattern of DdRACK1

**Figure 2:**
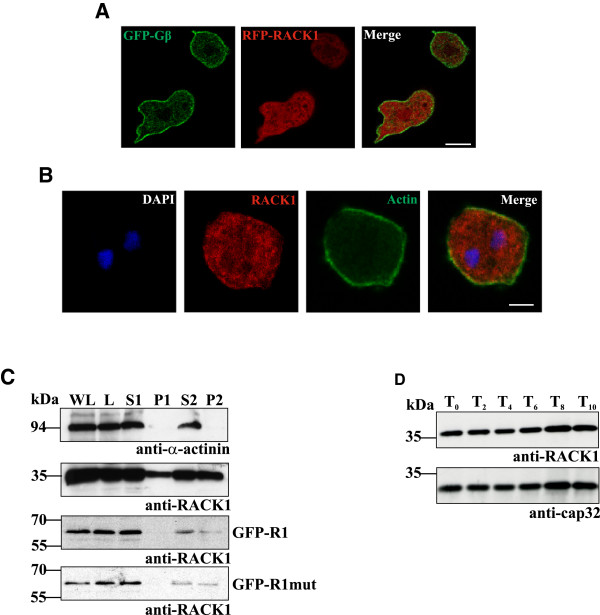
**Subcellular localization, and developmental level of expression of DdRACK1. (A)** To determine the localization of DdRACK1, AX2 wild type cells co-expressing GFP-Gβ and RFP-DdRACK1 were used to perform confocal live cell microscopy. **(B)** Immunofluorescence studies of AX2 wild type cells. Vegetative cells were fixed and stained with anti-DdRACK1 antibodies. mAb act1-7 against actin was used to visualize the cell cortex. Scale bar, 5 μm. **(C)** Subcellular fractionation of AX2 and AX2 expressing GFP-DdRACK1 and GFP-RACK1mut after lysis by passing through Nucleopore filters. Protein aliquots separated by SDS PAGE were used to perform western blot analysis. WL, whole cell lysate; L, supernatant from cell lysate (400 × g); S1, P1 (10,000 × g); S2, P2 (100,000 × g). S, supernatant; P, pellet. DdRACK1 and relative amount of GFP-DdRACK1 and GFP-DdRACK1mut were detected in supernatant as well as in pellet samples with polyclonal anti-DdRACK1 antibodies. mAb 47-16-8 detected the cytosolic marker protein α-actinin which served as control. The α-actinin blot for AX2 is shown. **(D)** DdRACK1 expression levels during development. Western blot analysis was performed with AX2 wild type cell samples collected during starvation in shaking suspension at indicated time points. DdRACK1 was detected with polyclonal anti-DdRACK1 antibodies. For loading control the blot was probed with mAb 188-19-95 which detects cap32.

When we expressed RFP-DdRACK1 in AX2 cells expressing the G protein beta-subunit as GFP-fusion protein for labeling the plasma membrane we found the protein present throughout the cytosol. A similar cytosolic RACK1 distribution was obtained when immunofluorescence studies were performed with AX2 cells stained with antibodies against DdRACK1 and actin for labeling the cell cortex (Figure 2A,B). On the other hand, immunofluorescence studies with aggregation competent AX2/GFP-Gβ cells stained with anti-DdRACK1 antibodies showed RACK1 enrichment at the cell periphery and also in cell protrusions (Additional file 1: Figure S1, S2 (arrow)). Interestingly, in highly polarized cells, DdRACK1 was enriched at the leading edge (Additional file 1: Figure S3, arrow).

In cell fractionation assays a significant amount of DdRACK1 was present in both the cytosolic and the pellet fraction. GFP-DdRACK1 and GFP-DdRACK1mut were also relatively present in pellet fractions. α-Actinin which served as cytosolic marker protein was exclusively present in the cytosolic fraction (Figure 2C). A membrane association of RACK1 is not surprising as it has been repeatedly found in phagosomal preparations from mouse and *Drosophila*, and GpbB has been found in phagosomal preparations from *D. discoideum*[42-45]. A developmental analysis showed the presence of DdRACK1 protein in nearly unaltered levels during all stages of *Dictyostelium* development (Figure 2D).

### Oligomerization potential of DdRACK1

**Figure 3:**
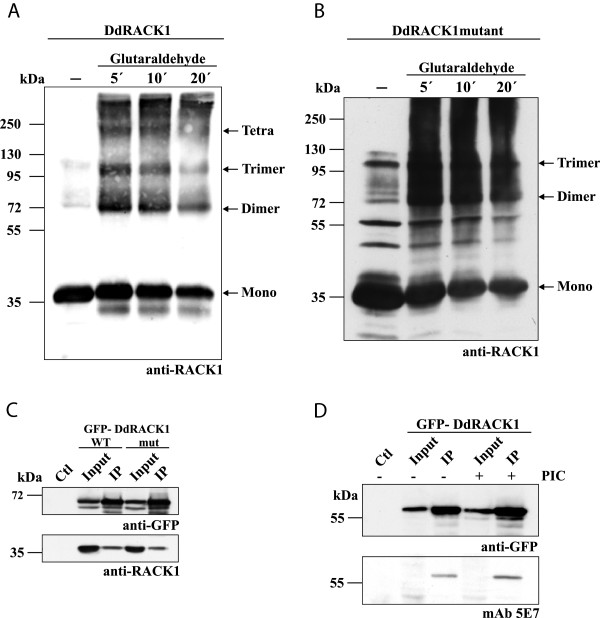
**DdRACK1 forms homodimers and oligomers and is a phosphotyrosine-containing protein. (A)** and **(B)** Analysis of DdRACK1 dimerization ability. 5–10 μg/100 μl of recombinant DdRACK1 **(A)** and DdRACK1mut **(B)** were incubated with 0.001% of the cross-linker glutaraldehyde and samples taken at the indicated time points of 5, 10 and 20 min. For both DdRACK1 and DdRACK1mut, in the absence of glutaraldehyde, the monomers (36 kDa, mono), including dimers (72 kDa), and trimers (108 kDa) were detected. Protein bands which correspond to tetramers were also detected for DdRACK1. Proteins were detected with polyclonal anti-DdRACK1 antibodies. **(C)** Co-immunoprecipitation analysis using GFP-DdRACK1 and GFP-DdRACK1mut. Both GFP-DdRACK1 and GFP-DdRACK1mut bound to GFP-trap beads (upper panel) were able to immunoprecipitate endogenous RACK1 (lower panel). For GFP-RACK1 fusions, degradation bands were observed. GFP-trap beads incubated with AX2 wild type cell lysate was used as control (Ctl). **(D)** Detection of DdRACK1 as a phosphotyrosine-containing protein. Western blot analysis was performed with proteins from immunoprecipitated GFP-DdRACK1 cell lysates (upper panel) prepared in presence (+) or absence (−) of phosphatase inhibitor cocktail (PIC). AX2 cell lysate incubated with GFP-trap beads was used as control (Ctl). The phosphotyrosine specific mAb 5E7 detected GFP-DdRACK1 in the IP (lower panel).

It has been suggested that RACK1 can dimerize in vivo and this dimerization is required for specific processes including the regulation of the *N*-methyl-D-aspartate (NMDA) receptor by the Fyn kinase in the brain [46-48,39,37]. Here, we tested the capability of DdRACK1 to oligomerize using recombinant DdRACK1 full length protein that had been cleaved from the GST part. In the presence of the cross-linking reagent glutaraldehyde (0.001%), DdRACK1 formed dimers and even higher oligomers with increasing time of incubation as detected by western blots using anti-DdRACK1 polyclonal antibodies. Interestingly, the native non-crosslinked DdRACK1 sample also contained some amount of dimers and oligomers (Figure 3A). This indicates that the dimerization characteristic exhibited by RACK1 proteins also holds true for DdRACK1. Similarly, DdRACK1mut also displayed wild type DdRACK1 oligomerization capability (Figure 3B). We further confirmed DdRACK1 dimerization by co-immunoprecipitation assays. Both GFP-DdRACK1 and GFP-DdRACK1mut bound to GFP-trap beads precipitated endogenous DdRACK1 (Figure 3C). AX2 cell lysates incubated with GFP-trap beads were used as control. Also, neither GFP bound to GFP-trap beads nor RFP bound to RFP-trap beads precipitated endogenous DdRACK1 (Figure 3C).

### Post-translational modification of DdRACK1

Little is known about post-translational modifications of RACK1 apart from phosphorylation which is emerging as an important factor that modulates the binding of proteins to RACK1. Phosphorylation of specific tyrosine residues and their corresponding functions has been reported [31,49-54]. To determine if DdRACK1 also possesses the potential of becoming phosphorylated, we enriched DdRACK1 by immunoprecipitating GFP-DdRACK1 from cell lysates that were prepared in the presence or absence of phosphatase inhibitor cocktail (PIC) and performed a western blot analysis using phosphotyrosine specific mAb 5E7 antibodies [55]. These antibodies recognized the GFP-DdRACK1 band on the blot indicating that DdRACK1, like RACK1 proteins from other species, can be phosphorylated on specific tyrosine residues (Figure 3D). AX2 cell lysate incubated with GFP-trap beads which was used as control showed no band.

### Lipid interactions

**Figure 4:**
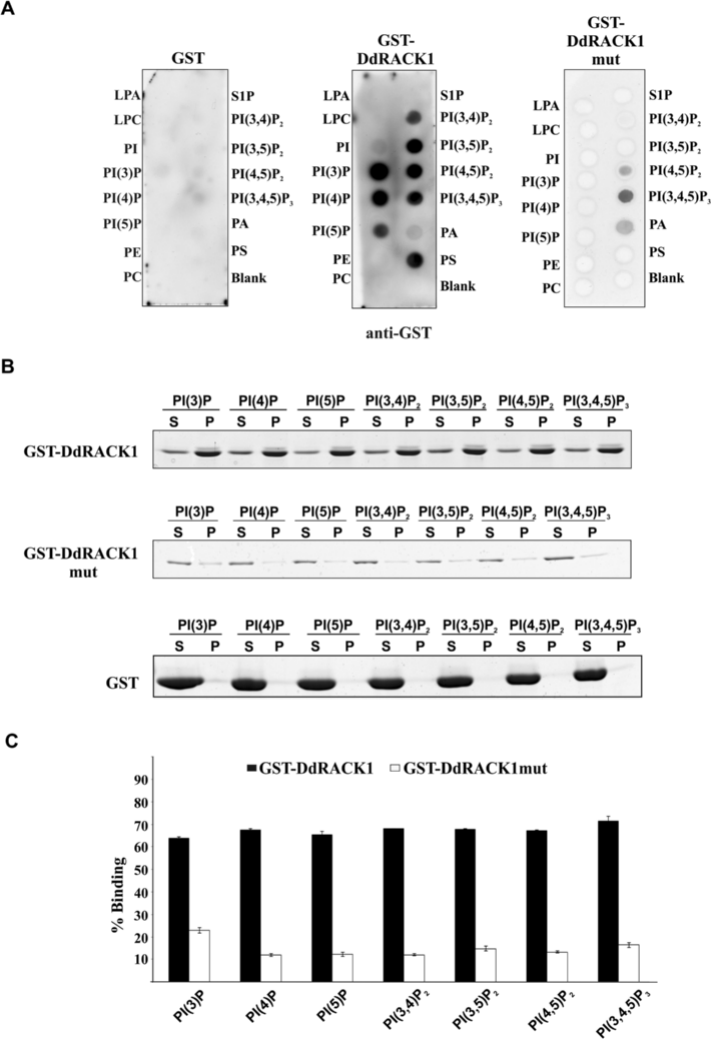
**DdRACK1 binds to phosphoinositides. (A)** PIP-Strip-membranes were incubated for 1 h at room temperature with 1 μg/ml GST (control), GST-DdRACK1 and GST-DdRACK1mut, respectively. Binding to PIPs on membranes was detected by incubation with polyclonal anti-GST antibodies. **(B)** Binding of GST-DdRACK1, GST-DdRACK1mut and GST (control) to PIPs in a liposome binding assay. 5–10 μg of GST and the GST-fusion proteins were incubated with liposomes containing 10% (wt/wt) of the indicated PIPs. Liposomes were collected by centrifugation, and bound proteins resolved by SDS-PAGE and detected by Coomassie Blue staining (S = supernatant; P = pellet). **(C)** Quantification of bound GST-DdARCK1 and GST-DdRACK1mut in pellet samples from **(B)**. Protein bands were quantified with ImageJ software.

The mechanism of membrane association of DdRACK1 is not known. In general, membrane association of proteins can be achieved by various mechanisms. For instance, polybasic clusters as defined by arginine- and lysine-enriched amino acid sequences enable diverse transmembrane and cytosolic proteins to bind lipids [56]. Also, proteins can target specific membranes through an interaction with phosphoinositides (PIPs). Based on the initial characterization of RACK1 as an interactor of RpkA, we tested the ability of DdRACK1 to bind to different phosphoinositides in vitro using GST-DdRACK1 in dot-blot (PIP strips) overlay assays. Whereas GST alone showed no PIP binding, GST-DdRACK1 bound with almost the same affinity to all the monophosphorylated PIPs, except for PI (3) P for which we observed stronger binding, to the bisphosphorylated PIPs as well as to the triphosphorylated PIP. GST-DdRACK1 also bound to phosphatidylserine (Figure 4A).

Although dot-blot overlay assays are convenient assays, they need to be supported by different methods as apparent specificities may be distorted and as they do not allow reliable quantification [57]. We therefore examined the sedimentation of GST-DdRACK1 with liposomes containing 65% phosphatidylcholine, 20% phosphatidylethanolamine, 5% phosphatidylserine, reconstituted with 10% individual phosphoinositides. Although without any specificity, GST-DdRACK1 showed significant binding to these liposomes indicating a broad binding specificity for membranes. GST was included as a control and did not sediment with the liposomes (Figure 4B). To quantitatively study to which PIPs DdRACK1 preferably bound, band intensities of the Coomassie blue stained gels were scanned and the pellet fractions plotted. This assay showed that DdRACK1 interacted equally well with all the different PIPs (Figure 4C).

The *A. thaliana* and *D. discoideum* RACK1 proteins carry an insertion between propeller blades 6 and 7, which contains primarily basic amino acids, in case of DdRACK1 six lysine residues (Figure 1A,C). By charge-reversal mutation analysis, the lysine residues (−KKKK-) were replaced with glutamic acid to generate a GST fusion mutant version of DdRACK1 (GST-DdRACK1mut). The mutant protein was used in dot-blot protein overlay assays where it still bound to PI (4,5) P_2_ and PI (3,4,5) P_3_, whereas binding to all other PIP variants was completely abolished (Figure 4A). In liposome sedimentation assays GST-DdRACK1mut did not show significant binding to any of the PIPs, which support the requirement of this polybasic region for lipid binding (Figure 4B, C).

### DdRACK1 interacts with G proteins

**Figure 5:**
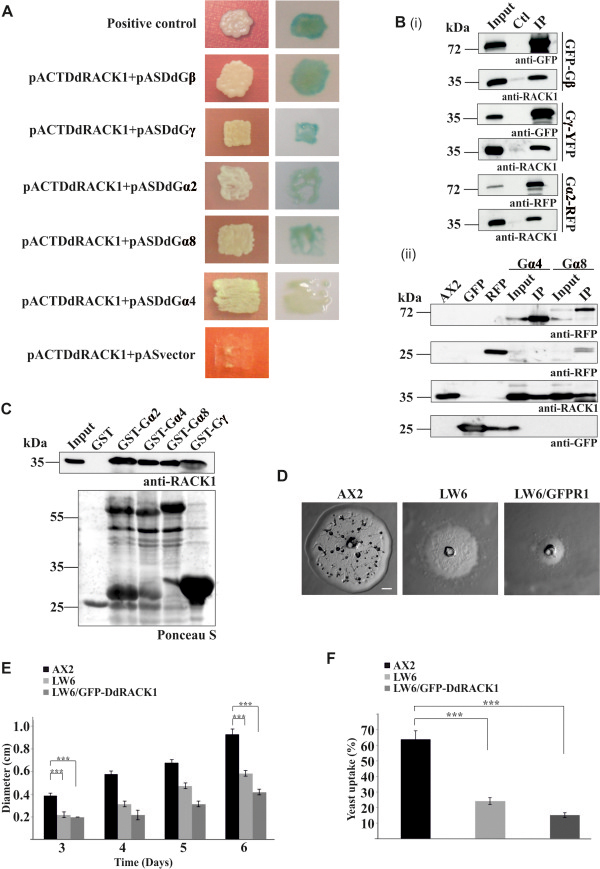
**DdRACK1 interacts with G proteins. (A)** Yeast two-hybrid analyses. Y190 strain was co-transformed with DdRACK1 in pACT2, and Gβ, Gγ, Gα2, Gα4, and Gα8 in pAS2, respectively. β-galactosidase activity was assayed for colonies grown on Pre-SD + 3AT agar plates. pACTDdNCAP + pASDdNCAP is positive, pACTDdRACK1 + pAS is negative control. Images were taken between 1 to 6 hours of staining with the exception of Gα4 for which the photograph was taken after 24 hours. **(B)** (i), Co-precipitation assays to confirm DdRACK1 interaction with the Gβ, Gγ and Gα2 protein subunits. GFP-Gβ, Gγ-YFP bound to GFP-trap beads and Gα2-RFP bound to RFP-trap beads precipitated endogenous DdRACK1 (IP). GFP-trap beads incubated with AX2 lysates were used as control (Ctl). (ii), Gα4-RFP and Gα8-RFP bound to RFP-trap beads co-precipitated endogenous DdRACK1 (IP). GFP and RFP bound to beads served as controls. mAb K3-184-2 detected GFP-tagged proteins, mAb K73-875-7 detected RFP-tagged proteins, polyclonal antibodies detected DdRACK1. **(C)** GST pulldown experiments to confirm DdRACK1 interaction with Gα subunits 2, 4 and 8, and Gγ, respectively. DdRACK1 was detected with polyclonal antibodies. The Ponceau S stained membrane is shown below to reveal the proteins employed in the pulldown. **(D)** Growth and development of AX2, *gβ* null mutants (LW6) and LW6/GFP-DdRACK1 cells on a *K. aerogenes* lawn. Images taken after 3 days are shown. Scale bar, 0.5 mm. **(E)** Measurement of plaque diameter to determine size of plaques formed by AX2, LW6 and LW6/GFP-DdRACK1 cells over several days. The bar represents the mean and SD of ten independent experiments (***P < 0.001). **(F)** Phagocytosis was assayed using the strains from **(E)** and TRITC-labelled yeast. Approximately 200 cells from each strain were counted. The percentage of cells which had engulfed yeast after 30 min is shown in the graph (***P < 0.001).

Conventional Gβ subunits exhibit a high affinity for Gγ subunits and function as Gβγ heterodimers to bind and stabilize GDP-bound Gα subunits. In addition, a Gβ can associate with multiple individual Gγ subunits [4]. The interaction of Gβγ with RACK1 was first identified by a yeast two-hybrid screen using the bovine Gβ1 sequence as bait to screen a mouse brain library [26]. To test whether DdRACK1 likewise associates with the *D. discoideum* Gβ and Gγ protein subunits, we performed a yeast two-hybrid analysis using DdRACK1 fused to the pACT2-AD. Gβ and Gγ subunits were fused to pAS2-BD, respectively. We detected interactions between DdRACK1 and Gβ as well as between DdRACK1 and Gγ subunits as revealed by β-galactosidase production (Figure 5A, blue staining of the colonies). Colonies from yeast transformed with pACTDdRACK1 and pAS vector did not grow on the selection plates. To confirm these interactions we performed co-immunoprecipitation analyses. GFP-Gβ as well as Gγ-YFP from AX2/GFP-Gβ and AX2/Gγ-YFP cell lysates, respectively, which were bound to GFP-trap beads, were able to independently co-immunoprecipitate endogenous DdRACK1 (Figure 5B (i)). AX2 wild type cell lysate incubated with GFP-trap beads was used as control (Ctl). GFP and RFP bound to beads did not immunoprecipitate DdRACK1 either (Figure 5B (ii)). This provides further evidence that DdRACK1 resembles RACK1 proteins and, like those, interacts with Gβ and Gγ subunits.

For *S. cerevisiae* it is reported that the RACK1 orthologue Asc1p functions as a Gβ subunit for a Gα (Gpa2) [30]. Similarly, in the human pathogenic fungus *C. neoformans* the RACK1 orthologue Gib2 functions as Gβ for Gpa1 [29]. Like *D. discoideum* both organisms have a single Gβ gene. To investigate DdRACK1-Gα interactions, yeast two-hybrid assays were performed using DdRACK1 fused to pACT2-AD. The Gα subunits Gα1, Gα2, Gα4, Gα5, Gα6, Gα7, Gα8, Gα9, Gα10, Gα11 and Gα12 were fused to pAS2-BD. In these assays, we detected stronger interactions between DdRACK1 and Gα2 as well as between DdRACK1 and Gα8, whereas a weak interaction was detected between DdRACK1 and Gα4 as concluded from the β-galactosidase staining assay (Figure 5A). Colonies from DdRACK1 interaction with the other Gα subunits analyzed did not grow on selection plates (Additional file 1: Figure S4). The DdRACK1 interactions with Gα 2, 4 and 8 were further confirmed in co-immunoprecipitation and pulldown experiments. Gα2-RFP, Gα4-RFP and Gα8-RFP bound to RFP-trap beads immunoprecipitated DdRACK1 from AX2 cell lysates, respectively (Figure 5B (i, ii)). Furthermore, GST-Gα2, GST-Gα4, GST-Gα8 as well as GST-Gγ pulled down endogenous DdRACK1 whereas GST did not (Figure 5C).

To analyze if DdRACK1 also takes over the Gβ function for the Gα subunits in vivo, we ectopically expressed DdRACK1 as a GFP fusion in the *gβ* null mutant LW6 [12,18] and analyzed whether it rescues the phagocytosis, chemotaxis, aggregation and developmental defects. We found that expression of GFP-DdRACK1 in LW6 cells did not rescue the developmental defect. When we plated the cells on a lawn of *K. aerogenes*, they formed smooth plaques as observed for the mutant strain. Remarkably the plaque size was even further reduced when we compared the AX2, LW6 and LW6/GFP-RACK1 strains (Figure 5D,E). This might be due to a further reduction in the rate of phagocytosis or enhanced defects in cell motility. Hence we examined their phagocytic capability following yeast particle uptake and found that whereas fewer LW6 cells had ingested one or more yeast particles after 30 min as expected when compared with AX2 cells, even fewer LW6/GFP-DdRACK1 cells took up yeast cells. Quantitatively, ~24% LW6 and ~15% LW6/GFP-DdRACK1 strains had taken up yeast cells as compared to ~64% uptake level for AX2 (Figure 5F). We conclude that Gβ functions are not taken over by RACK1 upon ectopic expression in *D. discoideum*.

*Dictyostelium* cells display an amoeboid type of cell motility. We performed single cell random migration assays with growth phase AX2, LW6 and LW6/GFP-DdRACK1 strains. Cells from all strains displayed similar motility with a speed of 6.62 ± 1.85 μm/min (AX2), 6.54 ± 3.53 μm/min (LW6) and 6.34 ± 1.95 μm/min (LW6/GFP-DdRACK1), respectively.

### Growth and development of *D. discoideum* strains

**Figure 6:**
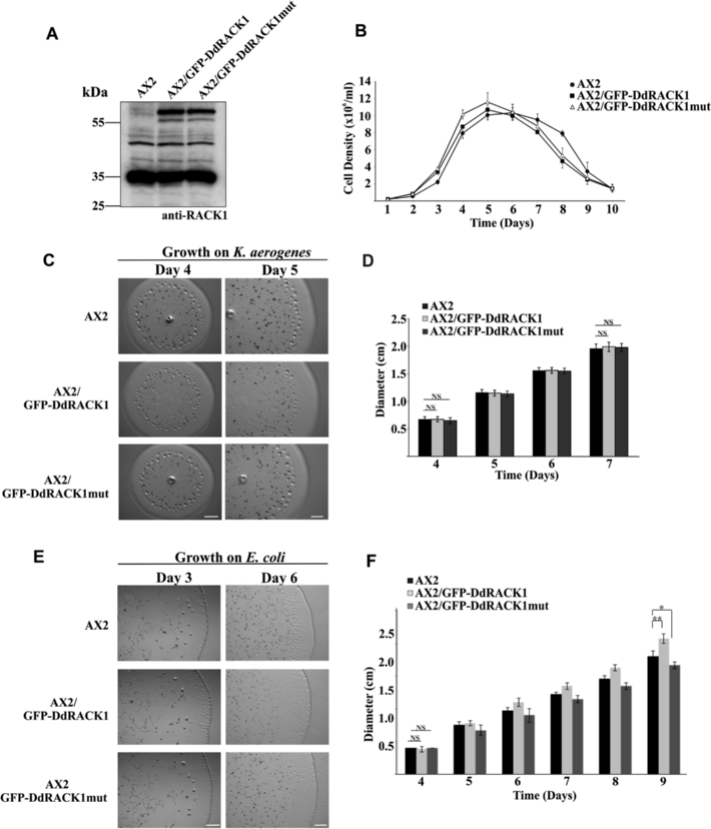
***D. discoideum *cells overexpressing DdRACK1 have growth and developmental defects. (A)** Levels of DdRACK1 overexpression. Cell lysates from vegetative AX2, AX2/GFP-DdRACK1 and AX2/GFP-DdRACK1mut strains were analyzed by SDS-PAGE and western blot. DdRACK1 and GFP-fusion proteins at 36 and 66 kDa were detected with polyclonal anti-DdRACK1 antibodies. **(B)** Growth in shaking suspension of *D. discoideum* strains. 5 x 10^4^cells/ml were used for inoculation. **(C)** Growth of *D. discoideum* strains on lawns of *K. aerogenes*. Images were taken on days 4 and 5. Scale bar, 1 mm. **(D)** Bar chart showing diameter of plaques formed by *D. discoideum* strains in **(C)** measured between days 4 and 7. The bar represents the mean and SD of ten independent experiments (NS, not significant; *P* > 0.05). **(E)** Growth of *D. discoideum* strains on lawns of *E. coli* B12 and imaged on days 3 and 6. Scale bar, 1 mm. **(F)** Bar chart showing diameter of plaques formed by *D. discoideum* strains in **(E)** measured between days 4 and 9. The bar represents the mean and SD of ten independent experiments (**P < 0.01; *P < 0.05; NS, not significant; *P* > 0.05).

Our attempts to generate either *D. discoideum* knockout and/or knockdown mutants for RACK1 using different molecular biology techniques were not successful. Since RACK1 acts as a scaffold protein, interference with its levels might lead to cellular defects which give an indication about its involvement in critical cellular roles. Knowing fully well that an overexpression of RACK1 has effects on various cell types [58-61], we therefore tried to also study the effects of RACK1 overexpression in a wild type background and characterized AX2 cells expressing GFP-DdRACK1 and GFP-DdRACK1mut. In western blot analysis with AX2, AX2/GFP-DdRACK1 and AX2/GFP-DdRACK1mut cells, we found that the levels of RACK1 with respect to GFP-RACK1 and endogenous RACK1 were only moderately enhanced (~17% in AX2/GFP-DdRACK1 and ~13% in AX2/GFP-DdRACK1mut cells, respectively) when the blot was probed with anti-DdRACK1 polyclonal antibodies (Figure 6A). Such a behavior may be the result of the scaffolding function. It has been proposed that the levels of scaffold proteins should be tightly regulated as misregulation might interfere with many cellular processes [62].

**Figure 7:**
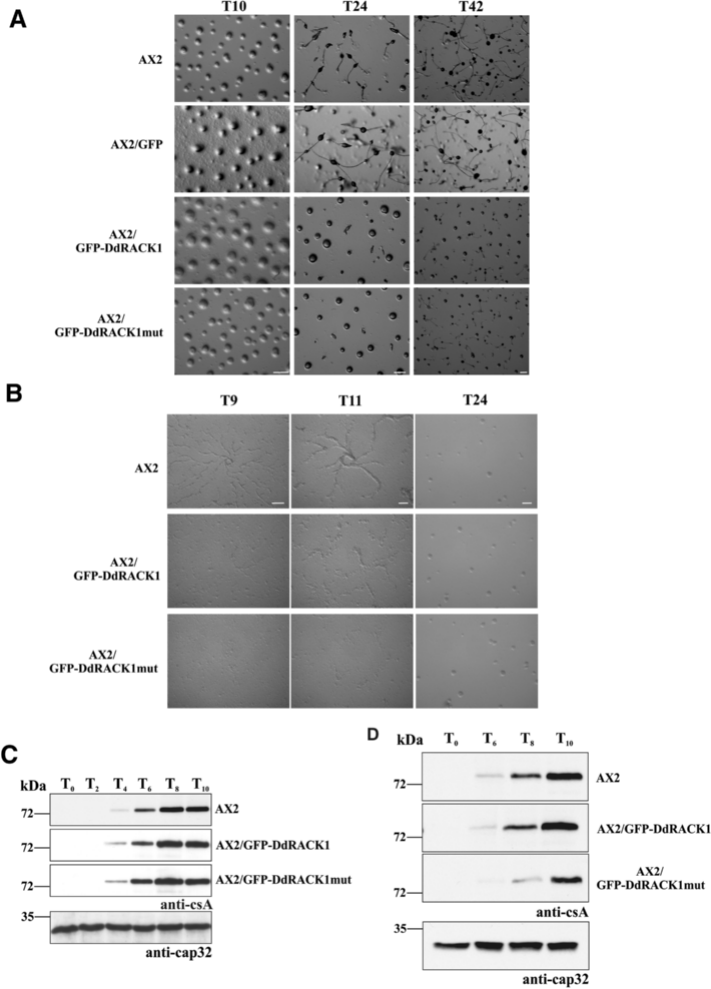
***D. discoideum *cells overexpressing DdRACK1 show delays and alterations in formation of developmental patterns. (A)** 5 × 10^7^ AX2, AX2/GFP, AX2/GFP-DdRACK1 and AX2/GFP-DdRACK1mut cells were deposited on phosphate agar plates and imaged at the indicated hours of development. Scale bar, 250 μm. **(B)** 1 × 10^7^*D. discoideum* strains were starved on petri dishes under phosphate buffer. Images were taken at the indicated hours. Scale bar, 250 μm. **(C)** Time-dependent expression of csA. Cells from *D. discoideum* were collected during development in shaking suspension at the indicated time points and analyzed by SDS-PAGE and western blot. csA was detected by mAb33-294, mAb 188-19-95 detected cap32 which was used as loading control. Since cap32 blot was similar for all strains, only the cap32 blot for AX2 is shown. **(D)** Cell samples of *D. discoideum* strains were collected from submerged cultures in phosphate buffer and tested for csA expression. The cap32 blot for AX2 is shown.

Growth in shaking suspension was comparable between AX2 and AX2 expressing GFP-DdRACK1 and GFP-DdRACK1mut with similar duplication times and similar final densities (~1 × 10^7^ cells/ml). However, once the cells had reached maximum density, AX2/GFP-DdRACK1 and AX2/GFP-DdRACK1mut cells did not stay in the stationary phase for long like AX2 as cell counts dropped rapidly (Figure 6B). Differences were also observed during growth on lawns of *K. aerogenes* on SM agar and *E. coli* B12 on nutrient agar (NA) plates. In these assays we noticed an expanded growth zone containing AX2/GFP-DdRACK1 amoebae when they were grown on *K. aerogenes* when compared with AX2 (Figure 6C). Upon growth on lawns of *E. coli* B12 the behavior for AX2/GFP-DdRACK1 strain was conspicuously different from AX2 on day 3 (Figure 6E). On *K. aerogenes* lawns, AX2 cells expressing GFP-DdRACK1mut were like wild type. AX2, AX2/GFP-DdRACK1 and AX2/GFP-DdRACK1mut displayed similar growth rates on lawns of *K. aerogenes* when the plaque diameter was measured between days 4 and 7 (Figure 6D). On *E. coli* lawns however, the AX2/GFP-DdRACK1 strain showed a significantly higher growth rate after 9 days whereas AX2 showed slightly increased growth compared to AX2/GFP-DdRACK1mut (Figure 6F). AX2 cells expressing GFP displayed growth behavior like AX2 wild type cells on a *K. aerogenes* lawn (Additional file 1: Figure S5). Faster growth on a bacterial lawn could be due to increased phagocytosis, altered cell motility or to a developmental defect.Therefore we next analyzed development which is initiated by starvation. AX2 cells plated on phosphate agar plates start to form multicellular aggregates between 8 to 12 hours and have formed fully differentiated fruiting bodies after ~24 hours. In our experiments cells from all strains had gathered into mounds at 10 hours. After 24 hours AX2 and AX2/GFP cells had formed fruiting bodies, whereas those of both AX2/GFP-DdRACK1 and AX2/GFP-DdRACK1mut were still present as tight aggregates and fruiting bodies which were much smaller than those of AX2 were observed only after 42 hours. There were still many mounds present (Figure 7A).

To investigate development further, we examined the aggregation behavior of these strains on a plastic surface. AX2 cells were highly elongated and formed well-defined streams after 9 hours of starvation. After 11 hours the streams became thicker and shorter. AX2/GFP-DdRACK1 and AX2/GFP-DdRACK1mut cells failed to form streams after 9 hours. AX2/GFP-DdRACK1 cells started to stream and form aggregates after 11 hours. Start of aggregate formation was even more delayed in AX2/GFP-DdRACK1mut cells (Figure 7B). An aggregation experiment with AX2/GFP cells revealed the same developmental pattern as seen for AX2 (Additional file 1: Figure S6). When we monitored the expression of the strictly developmentally regulated cell adhesion protein contact site A (csA) in cells starved in shaking suspension, we observed a similar expression pattern with a first detection after four hours of starvation and a steady increase in all three strains (Figure 7C). This was however not the case when we monitored csA expression from cells starved on plates. In AX2 and AX2/GFP-DdRACK1 csA was first detected after six hours of starvation, AX2/GFP-DdRACK1mut cells showed delayed csA expression with first detection after eight hours of starvation (Figure 7D) supporting the data obtained by visual inspection (Figure 7B).

## Discussion

Scaffold proteins uniquely integrate signals from multiple pathways. They generate lots of functional diversity by mediating a series of interactions with a vast array of protein partners. The receptor for activated C kinase 1 (RACK1) is a member of the evolutionarily conserved family of WD40 repeat proteins which forms seven β-propeller blades. It was initially discovered through its ability to function as a scaffold protein, bringing in close proximity protein kinase C (PKC) and its substrates [63,23]. In this study we report a novel protein in *D. discoideum* that is hitherto uncharacterized and displays significant homology with RACK1 proteins that have been well studied in various other species. Due to its high similarity to these other RACK1 proteins, we have named this protein DdRACK1.

DdRACK1 is a WD40 repeat protein harboring a seven-bladed β-propeller that shares similarities with the heterotrimeric G protein β subunit. The modelled structure of DdRACK1 features the seven β-propeller architecture with each propeller blade arranged in sequential order and made up of four-stranded antiparallel β-sheets. Although differences exist, particularly in the extended loop that connects β-propeller blades 6 and 7, the structures of RACK1 from *S. cerevisiae*[40], *A. thaliana*[39] and human [41] show significant sequence identity with DdRACK1. The region between the β-propeller blades 6 and 7 is quite conserved between DdRACK1 and *A. thaliana* RACK1A. The major difference between the WD repeats is in the loops that provide the distinct features of each member of the WD family and distinguish RACK1 interactions from those of other WD proteins [64,65]. The *A. thaliana* protein was the first RACK1 orthologue to be structurally described [31]. Unlike in *A. thaliana* where RACK1 is expressed by three genes, DdRACK1 is expressed by only one gene, *gpbB*, as in metazoans. Two conserved surface regions of *A. thaliana* RACK1A have been proposed to represent protein-protein interaction sites [39]. The first region is located on the top rim of the propeller and involves side chains from residues R36, K38, S63, H64 (blade 1), R42, K44, S70, H71; W83, D107 (blade 2), W90, D114; R125 (blade 3), R132; and W152 (blade 4), W158 in DdRACK1. The second large conserved surface region of RACK1 is located on the bottom of the propeller and is comprised of conserved residues P204, D205, Y230 (blade 5), P208, D209, Y234; and N246, Y248 and W249 (blade 6), N250, Y252 and W253 in DdRACK1. Besides the high sequence identity between DdRACK1 and RACK1 from other species, the presence and conservation of these above mentioned regions indicates that DdRACK1 is a member of the RACK1 family of WD40 repeats proteins and may undergo similar interactions.

Although DdRACK1 is mainly cytosolic as seen from live confocal microscopy pictures, immunofluorescence and fractionation studies, a portion of it was also found in the membrane fraction, buttressing localization to cellular membranes by proteins of the RACK1 family. Furthermore, DdRACK1 was detected at the cell periphery and the leading edge of highly polarized aggregation competent cells. This implies that RACK1 regulates signal transduction at the leading edge. RACK1 is essential for cell migration, and the protein binds to many components of the cell migration machinery including kinases, phosphatases and the cytoplasmic domains of cell surface receptors [65,66]. RACK1 is located in areas of cell protrusios that are rich in paxillin [67,68] and can increase the phosphorylation of FAK [68]. Furthermore, RACK1 has been reported to bind to components of the cytoskeleton [69,70]. Mutations in DdRACK1 did not seem to alter its localization to membranes as was observed from fractionation analysis in this study. One reason could be that it may have accompanied interaction partners to these sites.

RACK1 dimerizes both in vivo and in vitro [40,41,46]. The physiological role is however still unclear. In the regulation process of the NMDA receptor by Fyn, RACK1 dimerization is required to bring the two interacting partners in close contact. RACK1 dimerization allows exposing a new surface of the protein, buried within the propeller core in the monomeric form [46]. We have provided evidence that DdRACK1 also has the potential to dimerize. The dimerization of human RACK1 is enhanced by phosphorylation [47] and one of the putative phosphorylation sites was Ser146 in blade 3. This residue is however not conserved in DdRACK1, but there are other Ser/Thr residues present in this region which could probably be potential targets in mediating DdRACK1 dimerization by phosphorylation. On the other hand, an important factor which modulates the binding of RACK1 proteins to partners is tyrosine phosphorylation [31]. Phosphorylation/dephosphorylation of different tyrosine residues of human RACK1 regulates various cellular processes [49,50,71]. These tyrosine residues are also conserved in DdRACK1, and we provided evidence that the DdRACK1 protein is a phosphotyrosine-containing protein. However, this study did not associate the phosphorylation of DdRACK1 with a function.

Phosphoinositides (PIPs) regulate fundamental biological processes including cell growth and survival, membrane trafficking and cytoskeletal dynamics [72]. PIPs are tightly regulated during chemotaxis in *D. discoideum*, in particular, PI (3, 4, 5) P3 gradients are formed within the plasma membrane [73]. They are thought to be of differing importance for sensing of shallow and steep gradients [74,75]. In the region between β-propeller blades 6 and 7 we noted a key polybasic cluster (−KKKK-) in DdRACK1 which turned out to be responsible for binding to several PIPs; PI (3) P, PI (4) P, PI (5) P, PI (3, 4) P2, PI (3, 5) P2, PI (4, 5) P2, and PI (3, 4, 5) P3 without particular preference; and also to phosphatidylserine. The translocation of RACK1 from one subcellular location to another has been shown to mediate various cellular responses following a stimulus [25]. However, the mechanism of RACK1 localization to cellular membranes is not known. PIPs are clustered in distinct intracellular membranes and serve as marker for different organelles. We propose therefore that one way by which RACK1 localizes to different cellular membranes may be via its interaction with PIPs which in *D. discoideum* is mediated by the polybasic stretch. Whether RACK1 proteins from other species also interact with membrane lipids needs to be investigated.

**Figure 8:**
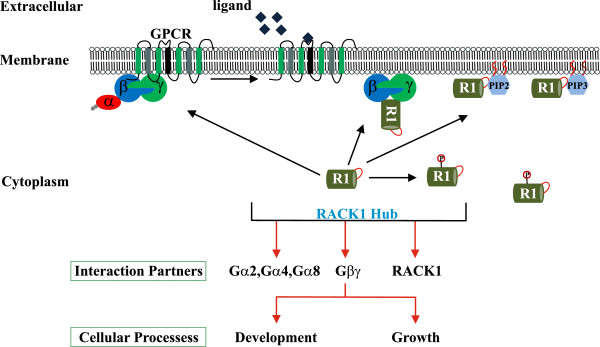
**Model for DdRACK1 involvement in growth and development.** The activation of Gβγ at the membrane initiates a series of events which modulates the production of PI (4,5) P_2_ and PI (3, 4, 5) P_3_ leading to the binding and recruitment of DdRACK1 (RI). DdRACK1 binds to G proteins (which may be in their free or heterotrimeric state). This association could regulate G protein interaction with downstream effectors. Phosphorylation/dephosphorylation of DdRACK1 further could regulate its cellular functions. Interaction with the different partners confers DdRACK1 with roles in various cellular processes like growth, motility and development.

G protein-linked signal transduction plays an essential role in the developmental program of *Dictyostelium*[76-78]. *D. discoideum* has twelve Gα subunits, one Gβ and one Gγ subunit. It is generally assumed that Gβ forms heterotrimers with the γ and all α subunits [79]. For RACK1, interactions with G protein heterotrimer and heterodimeric βγ subunits were reported [26-28]. We describe here an interaction of DdRACK1 with Gα subunits 2, 4 and 8, as well as with the Gβ and Gγ subunits by yeast two-hybrid, co-immunoprecipitation and pull down experiments. Whereas Gα subunits 2 and 4 are involved in chemotaxis, Gα8 was recently shown to function in cell proliferation, adhesion and cell differentiation. It is not very clear why DdRACK1 selectively interacts with these Gα subunits. However, RACK1 has been implicated in these cellular processes and the mutant phenotypes that we observed after overexpression revealed roles in cell growth and development. It further confers RACK1 with functions in the regulation of signaling processes in which these Gα subunits are involved (Figure 8). This also does not completely rule out the possibility of DdRACK1 interaction with the other Gα subunits which may be very weak to be detected by these approaches. Further studies still have to be done to determine structural mechanisms underlying DdRACK1 interaction with these Gα subunits.

## Conclusion

We have identified the novel RACK1 orthologue in *D. discoideum* (DdRACK1) which has significant sequence identity with other previously studied RACK1 species and similar biochemical features as bona fide RACK1 proteins. Together with the *Arabidopsis* protein it contains an unusual polybasic region through which it can bind to cellular membranes uncovering a further mechanism how RACK1 can be targeted to membranes. At the biochemical level it interacts with several proteins among them ribosomal proteins, enzymes, cytoskeletal proteins and most notably heterotrimeric G proteins. Upon overexpression we observe phenotypes that imply changes in signaling pathways regulated by the interacting G proteins. In summary (Figure 8), we propose that, through these interactions, RACK1 is involved in the regulation of several cellular processes.

## Materials and methods

### Growth, development and transfection

Cells were either grown on a lawn of *K. aerogenes* on SM agar plates, on a lawn of *E. coli* B12 on NA-agar or cultivated in shaking suspension (160 rpm) or in a submerged culture at 21-23°C in axenic medium [80]. Development was initiated by plating 5 × 10^7^ cells which were washed twice with Soerensen phosphate buffer (17 mM Na^+^/K^+^ phosphate, pH 6.0) on phosphate agar plates and monitored. Development was also followed for cells starved in Soerensen phosphate buffer in shaken suspension (1 × 10^7^ cells/ml; 160 rpm at 22°C) or in petri dishes. Mutants were maintained in the presence of appropriate antibiotics (2–4 μg/ml G418) (Roche Applied Science) (or 3–5 μg/ml Blasticidin) (MP Biomedicals Inc., Eschwege, Germany). The following strains have been used; AX2-214 (wild type) [81], AX2 expressing GFP-, YFP- or RFP-tagged fusion proteins, Gβ null mutants LW6 [12] and LW6 expressing GFP-DdRACK1. The corresponding plasmids were introduced by electroporation using a Biorad electroporator Gene Pulser Xcell (Biorad, München, Germany) according to the protocol supplied.

### Cloning of RACK1 cDNA and expression of recombinant proteins

For expression of recombinant *D. discoideum* RACK1 as glutathione S transferase (GST) fusion protein in *E. coli*, a full-length cDNA was cloned into pGEX-4 T-1 vector (GE Healthcare Life Sciences). *E. coli* strain XL1 Blue was used for expression of the GST fusion protein. Induction of protein expression was with 0.25 mM isopropyl β-D-thio-galactoside (IPTG) when an OD_600_ of 0.8 was reached. Cells were further cultured at 30°C for 3 hours. They were harvested, lysed in 50 mM Tris/HCl, pH 7.4 to 8.0, 100 mM NaCl, supplemented with Protease inhibitors (0.5 mM PMSF, 1 mM Benzamidine and Complete (Roche) and 1 mM DTT) with an EmulsiFlex cell homogenizer (Avestin Europe GmbH, Mannheim, Germany). Lysates were separated into soluble and insoluble fractions by centrifugation at 18,000 × g. The fusion proteins from the soluble fraction were purified using GST-Sepharose beads (GE Healthcare).

For cleavage of proteins from GST-Sepharose beads, the GST fusion proteins were washed 5 times with cleavage buffer (20 mM Tris/HCl, pH 7.4, 150 mM NaCl and 0.2% Sarcosyl). Beads were then resuspended in cleavage buffer and 3–10 U thrombin/mg fusion protein were added to the beads and incubated with little agitation at room temperature overnight. As RACK1 was released from the beads together with some GST, we next performed an anion exchange chromatography step in order to separate the proteins. For this the protein solution was dialyzed against 20 mM Tris/HCl, pH 8.0, and 1 mM EDTA overnight before loading onto a DE-52 Sephadex column which had been calibrated with 50 mM Tris/HCl, pH 8.0, 1 mM EDTA. The protein was eluted with 1 M NaCl and the eluate dialyzed and analyzed by SDS-PAGE.

For expression in AX2 the RACK1 cDNA was cloned into pBsr-N2-GFP vector (N-terminal GFP) and expressed as GFP-RACK1 under control of the actin 15 promoter and also into mRFPmars plasmid (N-terminal RFP) for RFP-RACK1 [82,83]. A PCR-mediated site-directed mutagenesis (QuikChange Site-Directed Mutagenesis Kit, Stratagene) was used to generate mutations in the GST-RACK1 and GFP-RACK1 plasmids. The mutations were confirmed by sequencing.

### Phosphoinositide binding assay

PIP-strips supplied by Echelon Biosciences, Inc. (Salt Lake City, Utah, USA) were used to perform phosphoinositide binding according to the supplied protocol. Briefly, GST and GST-fusion proteins were eluted from the glutathione agarose beads with elution buffer (20 mM reduced glutathione, 50 mM Tris/HCl, pH 7.4, 100 mM NaCl, 0.2% Tween-20, and 100 mM DTT).

The membranes were blocked with 0.1% ovalbumin (Sigma # A-5253) in TBS for one hour at room temperature. After discarding the blocking solution membranes were incubated with 1 mg/ml GST-fusion proteins in TBS-T (50 mM Tris/HCl, pH 7.4, 100 mM NaCl, 0.2% Tween-20) at room temperature for one hour. The protein solution was then discarded and the membranes were washed with TBS-T three times 10 minutes each. Bound protein was detected by western blot analysis with GST polyclonal antibodies as primary and anti-rabbit IgG-peroxidase (Sigma # A-6154) as secondary antibody followed by enhanced chemiluminescence.

### Lipid vesicle preparation and sedimentation assay

Phosphatidylserine (PS), phosphatidylcholine (PC), phosphatidylethanolamine (PE), PI (3) P, PI (4) P, PI (5) P, PI (3,4) P2, PI (3,5) P2, PI (4,5) P2, and PI (3,4,5) P3 were obtained from Sigma and diluted in chloroform. Liposome binding experiments were performed with a modified published liposome binding assay protocol [84]. Lipid mixtures containing 65% PC, 20% PE, 5% PS and 10% individual phosphoinositides were produced by mixing appropriate lipid solutions in chloroform/methanol. Slow flow nitrogen gas was used for the production of a film on the glass and vacuum desiccation for 30 min for solvent removal. Sterile-filtered sucrose binding buffer (20 mM HEPES, pH 7.4, 100 mM KCl, 1 mM EDTA, 0.1 M sucrose) was added to a final lipid concentration of 1 mg/ml and incubated at 37°C for 2 h. Lipids were then sonicated in a waterbath-sonicator for 10 sec. To test liposome binding, a 100 μl reaction mixture of freshly prepared liposomes and 5 μg of purified protein were incubated for 15 min at room temperature and centrifuged at 100,000 × g (42,000 rpm) at 4°C for 25 min in a Beckman table top ultracentrifuge Optima TLX (TLA 45 rotor). The supernatant was saved, and the pellet was resuspended in 100 μl of sucrose binding buffer.

Both fractions were then analyzed by SDS-PAGE followed by Coomassie blue staining. ImageJ was used for quantification.

### Yeast two-hybrid interaction

For the yeast two-hybrid screen, the full-length cDNAs of *D. discoideum* G protein β-, γ-, α1-, α2-, α4-, α5-, α6-, α7-, α8-, α9-, α10-, α11- and α12-subunits were cloned in frame into the yeast pAS2-1 vector (Clontech), respectively, resulting in fusion to the GAL4-DNA-BD (BD, binding domain). Full-length cDNA of DdRACK1 was cloned into the yeast pACT2 vector (Clontech) resulting in a fusion to the GAL4-DNA-AD (AD, activation domain). Yeast Y190 strain was used for this assay.

Candidate colonies expressing interacting proteins were screened by plating on SD/-Leu/-Trp/-His/+3AT plates after which membrane colonies-lift β-galactosidase activity assay was performed according to the MATCHMAKER Y2H system manual. Briefly, colonies on SD/-Leu/-Trp/-His/+3AT selection plates were transferred to a Nitrocellulose membrane (Protran BA 85) by placing the membrane over colonies on selection plates for 20 min. The filter was carefully lifted off the agar plates and transferred (with colonies facing up) to a pool of liquid nitrogen for 10 sec. The frozen filter was then allowed to thaw at room temperature and placed on a Whatman filter paper presoaked in freshly prepared X-Gal solution (60 mM Na_2_HPO_4_, 40 mM NaH_2_PO_4_, 10 mM KCl, 1 mM MgSO_4,_ pH 7.0, 50 mM β-mercaptoethanol, X-Gal (1 mg/ml final concentration)) and incubated at 30°C and checked between 1 to 6 h, and after 24 h (for detection of weak interactions) for the appearance of blue colonies.

### Pull down and immunoprecipitation assays

For pull down and immunoprecipitation experiments *D. discoideum* cells were lysed in 50 mM (10 mM for immunoprecipitation assay) Tris/HCl, pH 7.4, 150 mM NaCl, 0.5% NP40, supplemented with protease inhibitor cocktail (Sigma), 0.5 mM PMSF, 0.5 mM EDTA, and 1 mM Benzamidine by passing them through a 25G syringe (10–20 strokes) and incubated with agitation for 15 min at 4°C (to ensure complete cell lysis) followed by a centrifugation step at 16,000 rpm for 10 min. The supernatants were either incubated with GST and GST-fusion proteins, respectively, or with GFP-trap beads (ChromoTek, Martinsried, Germany). After incubation for 3 h GST beads were washed three times with wash buffer (50 mM Tris/HCl, pH 7.4, 150 mM NaCl, protease inhibitor cocktail, 0.5 mM PMSF, 0.5 mM EDTA, 1 mM Benzamidine), GFP-trap beads were washed with a different wash buffer (10 mM Tris/HCl, pH 7.4, 150 mM NaCl, protease inhibitor cocktail, 0.5 mM PMSF, 0.5 mM EDTA, 1 mM Benzamidine). The beads were resuspended in SDS sample buffer, incubated at 95°C for 5 min and the proteins separated by SDS-PAGE and analyzed by western blot. The Gβ and Gγ subunits used in this study were previously cloned into GFP (N-terminal) and YFP (C-terminal) vectors, respectively [85,13].

### In vitro cross-link assay

Purified DdRACK1 was used for a multimerization experiment as previously described [86]. Briefly, 5–10 μg/100 μl of RACK1 in 1 × PBS, pH 7.4, was incubated at room temperature in the presence of 0.001% (v/v) glutaraldehyde for various time points. The reaction was stopped by addition of glycine to a final concentration of 0.1 M after 5, 10 and 20 min, respectively. Samples were analyzed by SDS-PAGE and western blot.

### Test for presence of phosphotyrosine in DdRACK1

Samples from immunoprecipitation experiments from GFP-DdRACK1 bound to GFP-trap beads in the presence or absence of phosphatase inhibitors were analyzed by western blots and probed with anti-phosphotyrosine monoclonal antibody (5E7) [55].

### Immunofluorescence analysis and life cell imaging

Immunofluorescence study was performed as previously described [82]. Briefly, cells were transferred onto coverslips in Petri dishes and fixed by ice-cold methanol (5 min, 20°C). Cells were treated twice for 15 min (room temperature) with blocking solution (1× PBS containing 0.5% (wt/vol) BSA and 0.1% (vol/vol) fish gelatin). The appropriate antibodies were diluted in the blocking solution and applied on the cells for 1 h at room temperature; the excess of antibodies was removed by washing with the blocking solution before the 1 h incubation with the corresponding secondary antibodies. For live cell studies, cells were placed in 35 mm Petri dishes (ibidi GmbH-Martinsried, Germany) and allowed to adhere to the surface. Analysis of fixed and live cells was done by laser scanning confocal microscopy using a Leica TCS SP5 microscope equipped with a HyD detector.

### Cell migration studies

This analysis was done as previously described [73,82]. Briefly, growing cells were plated in a chamber (ibidi GmbH-Martinsried, Germany) and random motility was followed. Images were recorded at intervals of 6 s using a Leica DM-IL inverse microscope (Deerfield, IL; 40× objective) and a conventional CCD video camera and analyzed using Dynamic Image Analysis Software (DIAS, Soll Technologies, Iowa City, IA).

### Miscellaneous methods

Cell fractionation of AX2 cells was done as previously described [73]. Antibodies used in this study were mouse monoclonal antibodies mAb 47-16-8 directed against α-actinin [87], mAb 33–294 against the cell adhesion molecule csA [88], mAb 188-19-95 against the 32 kDa subunit of heterodimeric capping protein cap32/34 [89], mAb 5E7 against phosphorylated tyrosine residues [55], mAb K3-184-2 against GFP [90], mAb act1-7 against actin [91], mAb K73-875-7 against mRFPmars, rabbit polyclonal antibodies against GST [86]. Detection in western blots was with anti-mouse-IgG conjugated to peroxidase or peroxidase conjugated anti-rabbit-IgG antibodies.

For generation of rabbit polyclonal antibodies against DdRACK1, the GST-part of GST-DdRACK1 was removed by thrombin cleavage and DdRACK1 was used to immunize rabbits (Pineda, Berlin, Germany). The antibodies specifically recognized the bacterially produced recombinant protein, the RFP- and GFP-tagged fusion proteins as well as the endogenous protein in western blots of whole cell lysates; they were used in immunoprecipitation experiments as well as for immunofluorescence studies. Monoclonal antibody K73-875-7 was generated against bacterially expressed mRFPmars [83].

Protein sequences of RACK1 proteins from *H. sapiens* (P63244), *D. melanogaster* (O18640), *A. thaliana* (O24456), *S. cerevisiae* (P38011), and *D. discoideum* (P46800) were retrieved from Uniprot protein database and aligned using ClustalW program with Blosum 62 matrix. The aligned sequences were processed through EsPript for representation. The structural coordinates of *S. cerevisiae* RACK1 (Asc1p) was obtained from protein databank (PBD: 3FRX) (Figure 1B) and used as a template for modelling *D. discoideum* RACK1. MODELLER v9 was used to generate DdRACK1 model. Structures in Figure 1B and C were generated with the aid of the molecular visualization software PyMOL.

Experiments on animals followed internationally recognized guidelines and were approved by the authorities of the state of Northrhine-Westfalia.

## Competing interests

The authors declare that they have no competing interests.

## Authors’ contributions

NNO, KS and TYR planned and performed experiments. MP provided essential contributions to lipid binding and helped with the analysis, AMT provided essential reagents for antibody generation. AAN and TYR designed and supervised the study. NNO, AAN and TYR wrote the manuscript. All authors read and approved the final manuscript.

## Supplementary Material

Additional file 1: Figure S1 Distribution of endogenous DdRACK1 in AX2/GFP-Gβ cells. Some enrichment of RACK1 was seen at the cell periphery. Polyclonal RACK1 specific antibodies were used. Nuclei were stained with DAPI. Scale bar, 5 μm. **Figure S2.** Localization of DdRACK1 in aggregation competent AX2 cells. Aggregation competent AX2 cells formed extensions which are enriched for RACK1 (arrow). RACK1 was detected with polyclonal antibodies, actin with mAb act1-7. Nuclei were stained with DAPI. Scale bar, 5 μm. **Figure S3.** Localization of DdRACK1 in polarized cells. In this image, DdRACK1 was seen at the leading edge of polarized aggregation competent cells (arrow). Antibodies were as in Figure S2. Scale bar, 5 μm. **Figure S4.** Yeast two-hybrid analyses and β-galactosidase activity staining. Yeast Y190 strain that has *lacZ* and *His3* reporter genes was co-transformed with DdRACK1 in pACT2 vector and the Gα5, Gα6, Gα7, Gα9 and Gα12 protein subunits in pAS2 vector, respectively. Colonies did not grow on selection plates. **Figure S5.** Growth on lawns of *K. aerogenes* of AX2/GFP strain. Images of AX2/GFP strain on *K. aerogenes* lawns were taken between days 4 and 6. Plaque expansion was similar to that of AX2. Scale bar, 1 mm. **Figure S6.** Development of AX2/GFP strain on petri dishes under phosphate buffer. 1 × 10^7^ cells were starved on petri dishes and images taken at the indicated time points. The developmental behavior was similar to that of AX2. Scale bar, 250 μm. Click here for file
