# Supplementary material for: The Dictyostelium discoideum RACK1 orthologue has roles in growth and development
Source: Cell Commun Signal. 2014 Jun 15;12:37. doi: 10.1186/1478-811X-12-37 (PMC4094278; doi:10.1186/1478-811X-12-37)

S1

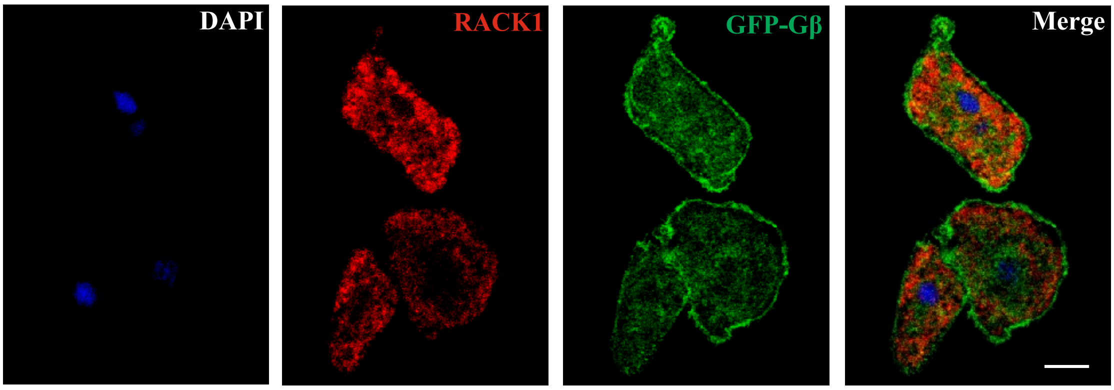

DAPI
RACK1
GFP-Gβ
Merge

S2

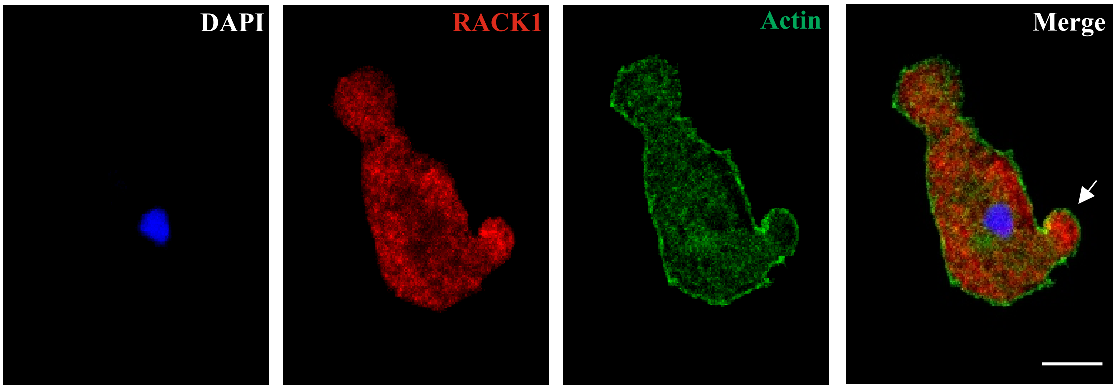

DAPI
RACK1
Actin
Merge

S3

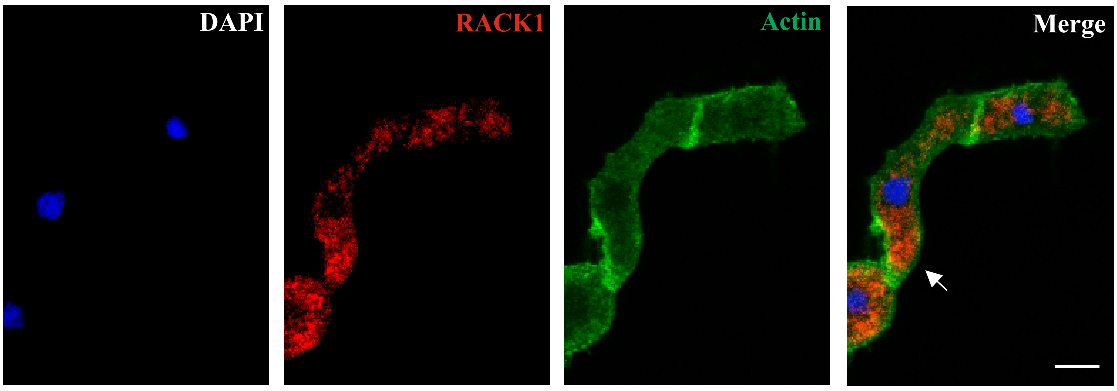

DAPI
RACK1
Actin
Merge

S4

pACTDdRACK1+pASDdGα5

pACTDdRACK1+pASDdGα6

pACTDdRACK1+pASDdGα7

pACTDdRACK1+pASDdGα9

pACTDdRACK1+pASDdGα12

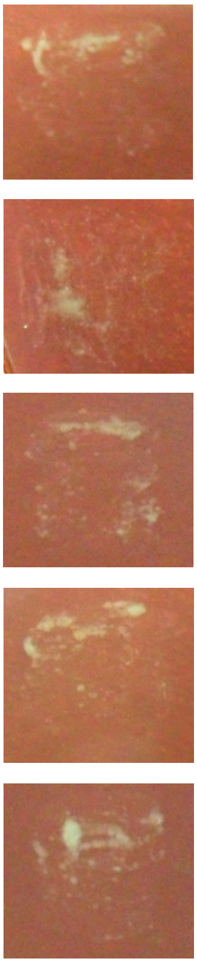

S5

Growth on *K. aerogenes*

Day 4 | Day 6

AX2/GFP

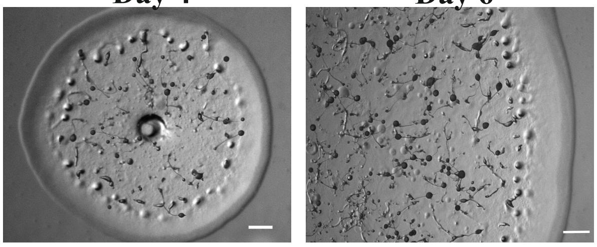

S6

T9 | T11 | T24

AX2/GFP

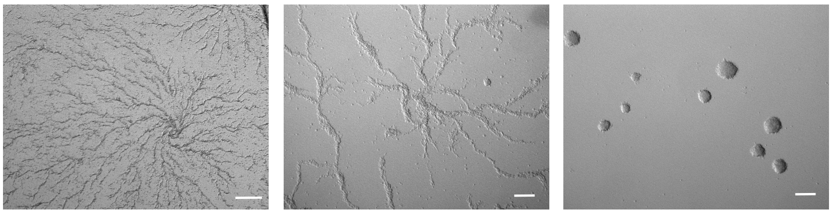

Supplement: Additional file 1: Figure S1 — Distribution of endogenous DdRACK1 in AX2/GFP-Gβ cells. Some enrichment of RACK1 was seen at the cell periphery. Polyclonal RACK1 specific antibodies were used. Nuclei were stained with DAPI. Scale bar, 5 μm. Figure S2. Localization of DdRACK1 in aggregation competent AX2 cells. Aggregation competent AX2 cells formed extensions which are enriched for RACK1 (arrow). RACK1 was detected with polyclonal antibodies, actin with mAb act1-7. Nuclei were stained with DAPI. Scale bar, 5 μm. Figure S3. Localization of DdRACK1 in polarized cells. In this image, DdRACK1 was seen at the leading edge of polarized aggregation competent cells (arrow). Antibodies were as in Figure S2. Scale bar, 5 μm. Figure S4. Yeast two-hybrid analyses and β-galactosidase activity staining. Yeast Y190 strain that has lacZ and His3 reporter genes was co-transformed with DdRACK1 in pACT2 vector and the Gα5, Gα6, Gα7, Gα9 and Gα12 protein subunits in pAS2 vector, respectively. Colonies did not grow on selection plates. Figure S5. Growth on lawns of K. aerogenes of AX2/GFP strain. Images of AX2/GFP strain on K. aerogenes lawns were taken between days 4 and 6. Plaque expansion was similar to that of AX2. Scale bar, 1 mm. Figure S6. Development of AX2/GFP strain on petri dishes under phosphate buffer. 1 × 10^7^ cells were starved on petri dishes and images taken at the indicated time points. The developmental behavior was similar to that of AX2. Scale bar, 250 μm. [file 1478-811X-12-37-S1.pdf]
